# DNA repair‐deficient premature aging models display accelerated epigenetic age

**DOI:** 10.1111/acel.14058

**Published:** 2023-12-22

**Authors:** Kevin Perez, Alberto Parras, Sara Picó, Cheyenne Rechsteiner, Amin Haghani, Robert Brooke, Calida Mrabti, Lucas Schoenfeldt, Steve Horvath, Alejandro Ocampo

**Affiliations:** ^1^ Department of Biomedical Sciences, Faculty of Biology and Medicine University of Lausanne Lausanne Switzerland; ^2^ EPITERNA SA Epalinges Switzerland; ^3^ Altos Labs San Diego California USA; ^4^ Epigenetic Clock Development Foundation Torrance California USA; ^5^ Human Genetics, David Geffen School of Medicine University of California Los Angeles California USA

**Keywords:** accelerated aging, DNA damage, epigenetic clock, methylation, progeria

## Abstract

Several premature aging mouse models have been developed to study aging and identify interventions that can delay age‐related diseases. Yet, it is still unclear whether these models truly recapitulate natural aging. Here, we analyzed DNA methylation in multiple tissues of four previously reported mouse models of premature aging (*Ercc1*, *LAKI*, *Polg*, and *Xpg*). We estimated DNA methylation (DNAm) age of these samples using the Horvath clock. The most pronounced increase in DNAm age could be observed in *Ercc1* mice, a strain which exhibits a deficit in DNA nucleotide excision repair. Similarly, we detected an increase in epigenetic age in fibroblasts isolated from patients with progeroid syndromes associated with mutations in DNA excision repair genes. These findings highlight that mouse models with deficiencies in DNA repair, unlike other premature aging models, display accelerated epigenetic age, suggesting a strong connection between DNA damage and epigenetic dysregulation during aging.

## Graphical abstract

Despite decades of research, it is still unclear whether mouse models of premature aging truly recapitulate physiological aging. Analyzing DNA methylation in four such models, we found that *Ercc1* and *Xpg* mice, deficient in DNA repair, exhibited the most significant increase in epigenetic age. Similarly, fibroblasts from progeroid syndrome patients with DNA repair gene mutations showed accelerated epigenetic aging. These results suggest a strong link between DNA damage and epigenetic dysregulation during aging.

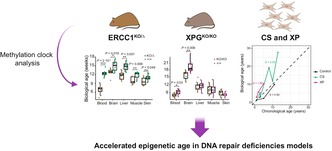

**Abbreviations**
CSA: Cockayne Syndrome type A
CSB: Cockayne Syndrome type B
DNAm: DNA methylation
LAKI: LMNA knock‐in
NER: nucleotide excision repair
XP: Xeroderma Pigmentosum

## 1 INTRODUCTION

The world's population is growing older. Since aging represents the strongest risk factor for most human diseases, it is therefore key to identify anti‐aging interventions that could delay or reverse the aging process (Partridge et al., 2018). Towards this goal, several accelerated aging mouse models have been developed (Koks et al., 2016; Liao & Kennedy, 2014). Premature aging models could speed up the discovery of anti‐aging interventions, nevertheless, their physiological relevance and whether they truly recapitulate or phenocopy natural aging remains controversial. With the recent development of biological aging clocks, epigenetic marks can now accurately predict age in multiple tissues in mammals (Ake Lu et al., 2021; Bell et al., 2019; Bergsma & Rogaeva, 2020; Horvath, 2013; Simpson & Chandra, 2021). Interestingly, several anti‐aging interventions have been shown to retard these clocks (Field et al., 2018), including cellular reprogramming (Browder et al., 2022; Lu et al., 2020).

## 2 DNA REPAIR DEFICIENT MOUSE MODELS EXHIBIT ACCELERATED EPIGENETIC AGE

**FIGURE 1:**
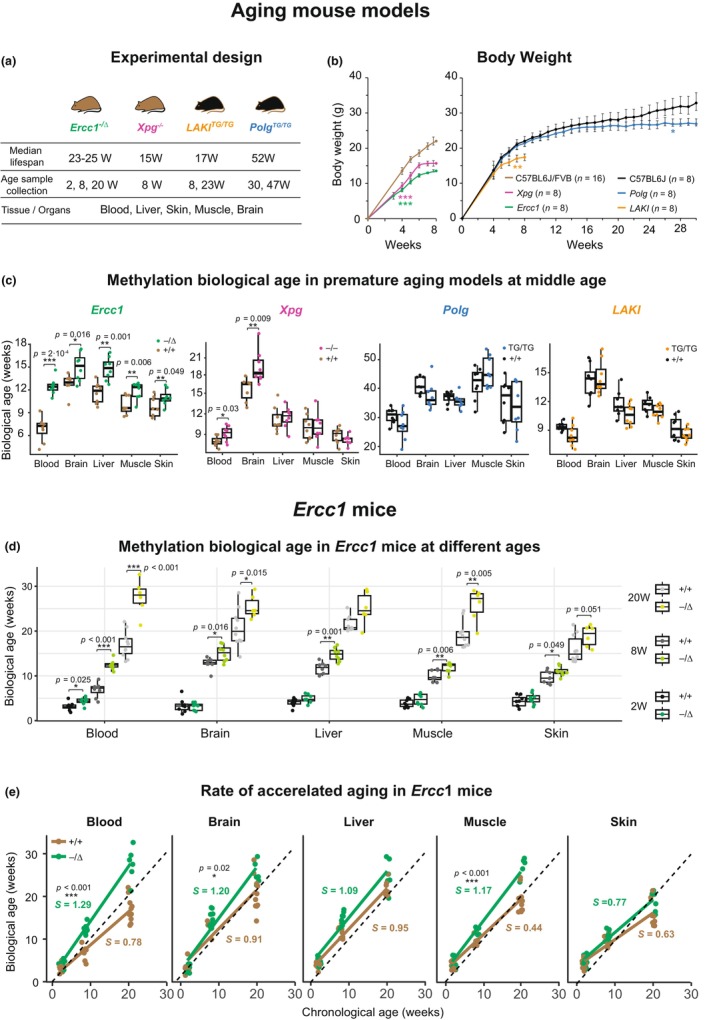
(a) Schematic representation of premature mouse strains, tissues collected, and timepoints taken. (b) Evolution of body weight until the euthanize point. (c) Methylation biological age of *Ercc1* ^*−/Δ*^, *Xpg* ^*−/−*^, *LAKI* ^*TG/TG*^, and *Polg* ^*TG/TG*^ mice. (d) Methylation biological age of *Ercc1* ^*−/Δ*^ mice at 2, 8, and 20 weeks in multiple organs/tissues. Data are represented as box plots (center line shows median, box shows 25th and 75th percentiles and whiskers show minimum and maximum values), statistical significance was assessed by two‐sided unpaired *t*‐test. (e) Slope of aging in *Ercc1* ^*−/Δ*^ and controls mice from 2 to 20 weeks old. Significance of the interaction term in the linear regression was analyzed.

Here, to assess the relevance of several premature aging mouse models (*Ercc1*, *Xpg*, *LAKI* and *Polg* mice), we analyzed the epigenetic age of multiple tissues using a DNA methylation clock, known as the “Horvath Pan Tissue clock” (Mozhui et al., 2022). In these mice, various biological mechanisms are thought to cause premature aging; ERCC1 (Weeda et al., 1997) and XPG (Barnhoorn et al., 2014) mice exhibit a deficit in nucleotide excision repair (NER) of the nuclear DNA, POLG mice show accumulation of mitochondrial DNA mutations (Kujoth et al., 2005; Trifunovic et al., 2004) and LMNA knock‐in (LAKI) mice suffer from nuclear lamina defects (Osorio et al., 2011; Varga et al., 2006). Here, we assessed DNA methylation age (DNAm) in *Ercc1* ^*−/Δ*^, *Xpg* ^*−/−*^, *Laki* ^*TG/TG*^, and *Polg* ^*TG/TG*^ mice at different ages, including post‐natal development, and middle and old age. Both proliferative (blood and skin) and more terminally differentiated tissues (liver, cerebral cortex and skeletal muscle) were analyzed (Figure 1a).

During the generation of experimental mice, we noticed that while *LAKI* ^*TG/TG*^ and *Polg* ^*TG/TG*^ mice were born at a predicted Mendelian frequency, *Ercc1* ^*−/Δ*^ and *Xpg* ^*−/−*^ showed a perinatal lethality (Figure S1a). Furthermore, all premature aging animals exhibited reduced body weight compared to their control littermates as expected (Figure 1b). As a quality check, we first looked at the clock performance in the control WT mice in multiple tissues. The chronological age prediction was highly accurate in blood in C57BL6J and C57BL6J‐FVB backgrounds (*r* = 0.99 and *r* = 0.95, respectively) and provided sufficient accuracy in the other tissues (*r* = 0.89 to 0.98) (Figure S1b and Table S1), confirming the precision of DNAm clocks. Next, we determined DNAm age in *Ercc1* ^*−/Δ*^, *LAKI* ^*TG/TG*^, and *Xpg* ^*−/−*^ at 8 weeks, and *Polg* ^*TG/TG*^ at 30 weeks of age corresponding to the relative middle age of the strain. Importantly, the biological age of *Ercc1* ^*−/Δ*^ mice was mainly increased in blood but also significantly increased in brain, liver, skeletal muscle, and skin according to the pan‐tissue or tissue‐specific clocks (Figure 1c and Table S4 respectively). Additionally, *Xpg* ^*−/−*^ mice showed increased age in blood and brain (Figure 1c). Conversely, we did not detect systemic DNAm age acceleration in LAKI or *Polg* mice in any of the tissues analyzed (Figure 1c). Additionally, we performed the “Tissue specific clock” analysis in mouse samples and we found similar results (Table S4).

Subsequently, we analyzed the methylation age at different ages including, *Ercc1* ^*−/Δ*^ (2, 8 and 20 weeks), *LAKI* ^*TG/TG*^ (8 and 23 weeks), and *Polg* ^*TG/TG*^ (30 and 47 weeks). Interestingly, in the *Ercc1* ^*−/Δ*^ mice, biological age was increased mildly at 2 weeks old in blood, and significantly accelerated in liver, and skin at 20 weeks (Figure 1d and Table S2). Conversely, DNAm age was not changed in aged *LAKI* ^*TG/TG*^ or *Polg* ^*TG/TG*^ mice (Figure S1c). Together, our results further confirm that biological age is increased only in *Ercc1* mice, at multiple ages.

Next, to determine if the rate of accelerated aging in *Ercc1* mice was constant or increasing with age, we calculated the slope of biological vs. chronological age in both WT and KO mice. Importantly, the rate was significantly increased in blood, skeletal muscle, and brain (Figure 1e), demonstrating that the delta between biological and chronological age increased with age in *Ercc1* ^*−/Δ*^ mice. All together, these results suggest DNA repair deficient mice as perhaps as one of the most promising mouse models of premature aging.

## 3 INCREASED DNAm AGE IN HUMAN CELLS FROM PROGEROID SYNDROMES

**FIGURE 2:**
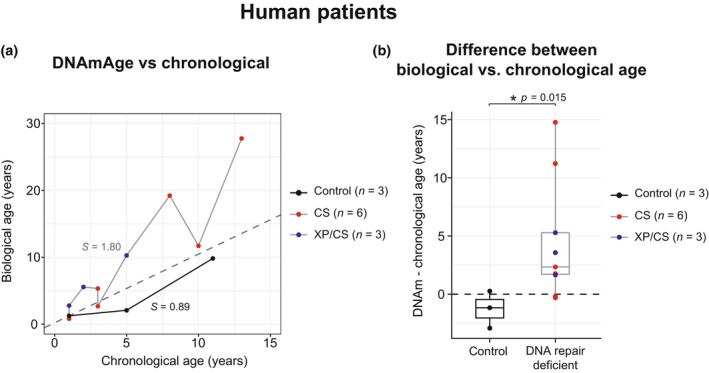
(a) DNAm age versus chronological age and (b) difference between biological and chronological age in control versus Cockayne Syndrome (CS) and Xeroderma Pigmentosum fibroblasts isolated from human samples. Data are represented as box plots (center line shows median, box shows 25th and 75th percentiles and whiskers show minimum and maximum values), statistical significance was assessed by two‐sided unpaired *t*‐test.

At last, to determine whether these findings also apply to humans, we analyzed the DNAm age of human fibroblasts obtained from patients affected by diseases caused by mutations in DNA excision repair genes associated with aging phenotypes: Xeroderma Pigmentosum (XP) (Rizza et al., 2021), and Cockayne Syndrome type A (CSA) and B (CSB) (Laugel, 2000) (Table S3). DNAm age was significantly higher in the affected patients (Figure 2a), and the difference between DNAm age and chronological age was significantly increased (Figure 2b). These results indicate that human progeroid syndromes associated with mutations in DNA excision repair genes also display accelerated epigenetic age.

Although premature aging models have been widely used, their physiological relevance from the perspective of aging clocks has not been deeply investigated until now. Here, we observed accelerated epigenetic aging in *Ercc1/5*‐deficient mice, and in related human diseases. Depletion of these proteins results in a defect in DNA repair, leading to an accumulation of DNA mutations. Our results indicate that defective DNA repair, leading to unrepaired persistent DNA damage, results in accelerated epigenetic aging, strongly suggesting a link between DNA damage and epigenetic dysregulation. In this line, DNA damage has been proposed as one of the central hallmarks of aging, as well as a potential driver of the aging process (Schumacher et al., 2021). We noted that even though DNAm age was increased in *Ercc1* mice already at 2 weeks, greater changes were observed in older animals indicating a progressive age acceleration during aging. In this line, we propose that a higher DNA repair capacity during development (Mitchell & Hartman, 1990), might prevent potential epigenetic dysregulation as consequence of DNA damage, and could protect these mutant mice during gestation. Interestingly, dietary restriction, shown to have a preventing impact on aging, dramatically extends lifespan of DNA repair‐deficient mice (Vermeij et al., 2016) and *Ercc1* depletion in blood specifically causes premature aging (Yousefzadeh et al., 2021).

Importantly, the methylation clock was more accurate in blood, a rapidly proliferative tissue that undergoes constant regeneration, in which the most significant and strongest differences were observed in DNA repair deficient mice. Therefore, due to its simple collection and strong sensitivity for detection of epigenetic aging, we propose the use of blood as one of the best tissues to study and analyze the effect of anti‐aging interventions. Additionally, the age acceleration observed in CS patients is in line with other biomarkers previously used to predict biological age such as GlycoAgeTest (Vanhooren et al., 2010). At last, while multiple groups have examined the biological age of human diseases associated with premature aging and no changes in DNAm age have been observed in the blood of Hutchinson‐Gilford progeria syndrome patients (Bejaoui et al., 2022), a significant increase in biological age was observed in multiple human genetic disorders such as Werner (Maierhofer et al., 2017), Down (Xu et al., 2022) Sotos (Martin‐Herranz et al., 2019), Tatton‐Brown‐Rahman (Jeffries et al., 2019), Leigh (Yu et al., 2022) syndromes.

## 4 IN SUMMARY

Our survey of mouse models of premature aging may be expanded to alternative models (Koks et al., 2016), additional tissues, and ages. Moreover, since one limitation of our study might be the use of the Horvath pan‐tissue clock mainly, it would be interesting to also assess biological age using other epigenetic clocks, as well as other clocks built on different types of omics data, such as transcriptomic, proteomic or chromatin accessibility clocks (Lehallier et al., 2020; Meyer & Schumacher, 2021; Rechsteiner et al., 2022).

## AUTHOR CONTRIBUTIONS

K.P. and S.P. performed data and statistical analysis. A.P. generated mouse strains and collected tissues. C.M and L.S. were involved in culture and DNA extraction from human cells. C.R. extracted DNA from mice. S.H., A.H. analyzed data and made a critically revision. A.O. directed and supervised the study. K.P., A.P, and S.P. wrote the manuscript with input from all authors.

## CONFLICT OF INTEREST STATEMENT

K.P. and A.O. are co‐founders and shareholders of EPITERNA SA (non‐financial interests). A.O. is co‐founder of Longevity Consultancy Group (non‐financial interests). S.H. is a founder of the non‐profit Epigenetic Clock Development Foundation, which licenses several patents from his former employer UC Regents. These patents list S.H. as inventor. The rest of the authors declare no competing interests.

## Supporting information


Appendix S1

## Data Availability

The data that supports the findings of this study are available in the supplementary material of this article.
