# Supplementary material for: DNA repair‐deficient premature aging models display accelerated epigenetic age
Source: Aging Cell. 2023 Dec 22;23(2):e14058. doi: 10.1111/acel.14058 (PMC10861193; doi:10.1111/acel.14058)
Supplement: Supplementary file 1 — Appendix S1 [file ACEL-23-e14058-s001.zip › acel14058-sup-0001-AppendixS1.pdf]

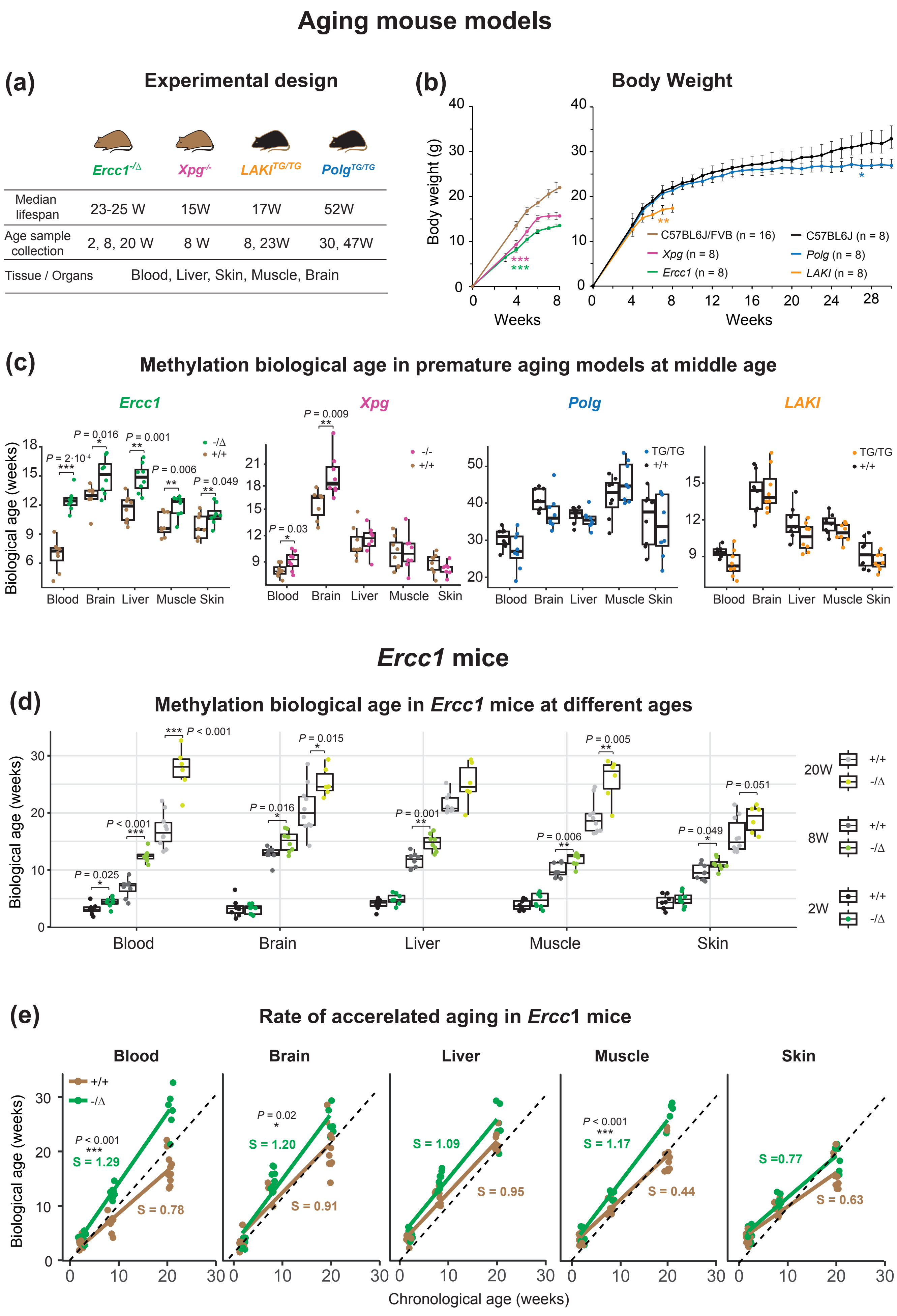

Aging mouse models
(a)
Experimental design
Ercc1-/Δ
Xpg-/-
LAKITG/TG
PolgTG/TG
Median lifespan
23-25 W
15W
17W
52W
Age sample collection
2, 8, 20 W
8 W
8, 23W
30, 47W
Tissue / Organs
Blood, Liver, Skin, Muscle, Brain
(b)
Body Weight
Body weight (g)
Weeks
C57BL6J/FVB (n = 16)
Xpg (n = 8)
Ercc1 (n = 8)
C57BL6J (n = 8)
Polg (n = 8)
LAKI (n = 8)
(c)
Methylation biological age in premature aging models at middle age
Ercc1
Xpg
Polg
LAKI
Biological age (weeks)
Blood Brain Liver Muscle Skin
Ercc1 mice
(d)
Methylation biological age in Ercc1 mice at different ages
Biological age (weeks)
Blood
Brain
Liver
Muscle
Skin
20W
8W
2W
(e)
Rate of accelerated aging in Ercc1 mice
Blood
Brain
Liver
Muscle
Skin
S = 1.29
S = 0.78
S = 1.20
S = 0.91
S = 1.09
S = 0.95
S = 1.17
S = 0.44
S =0.77
S = 0.63
Biological age (weeks)
Chronological age (weeks)

Figure 1

Human patients

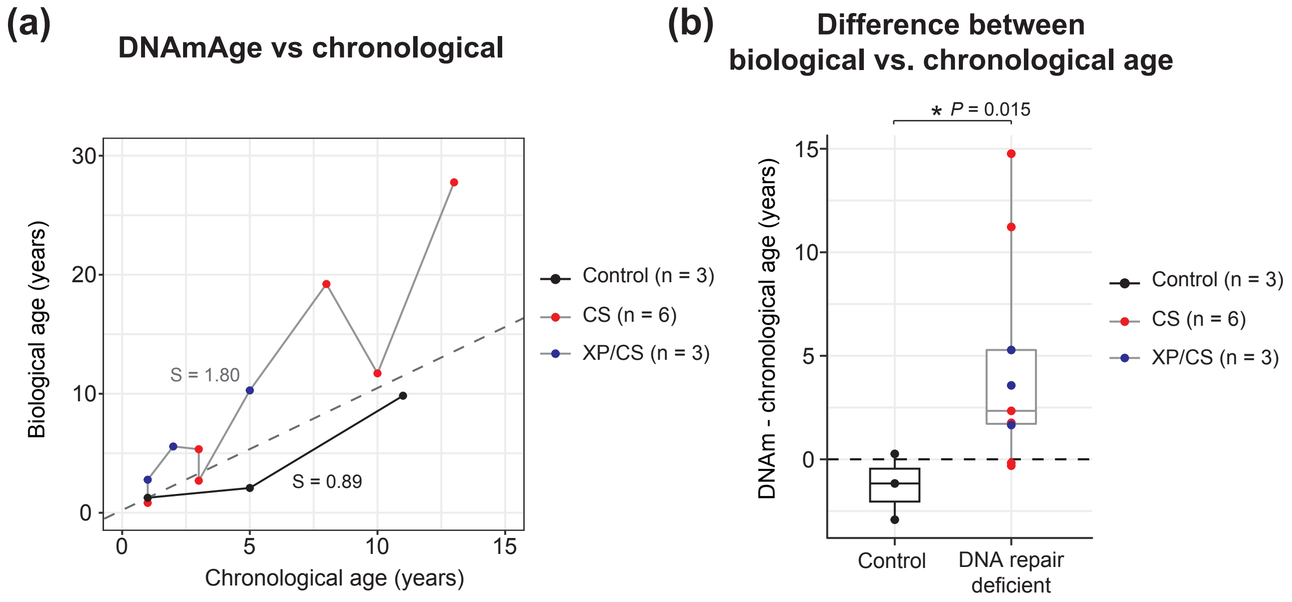

Figure 2

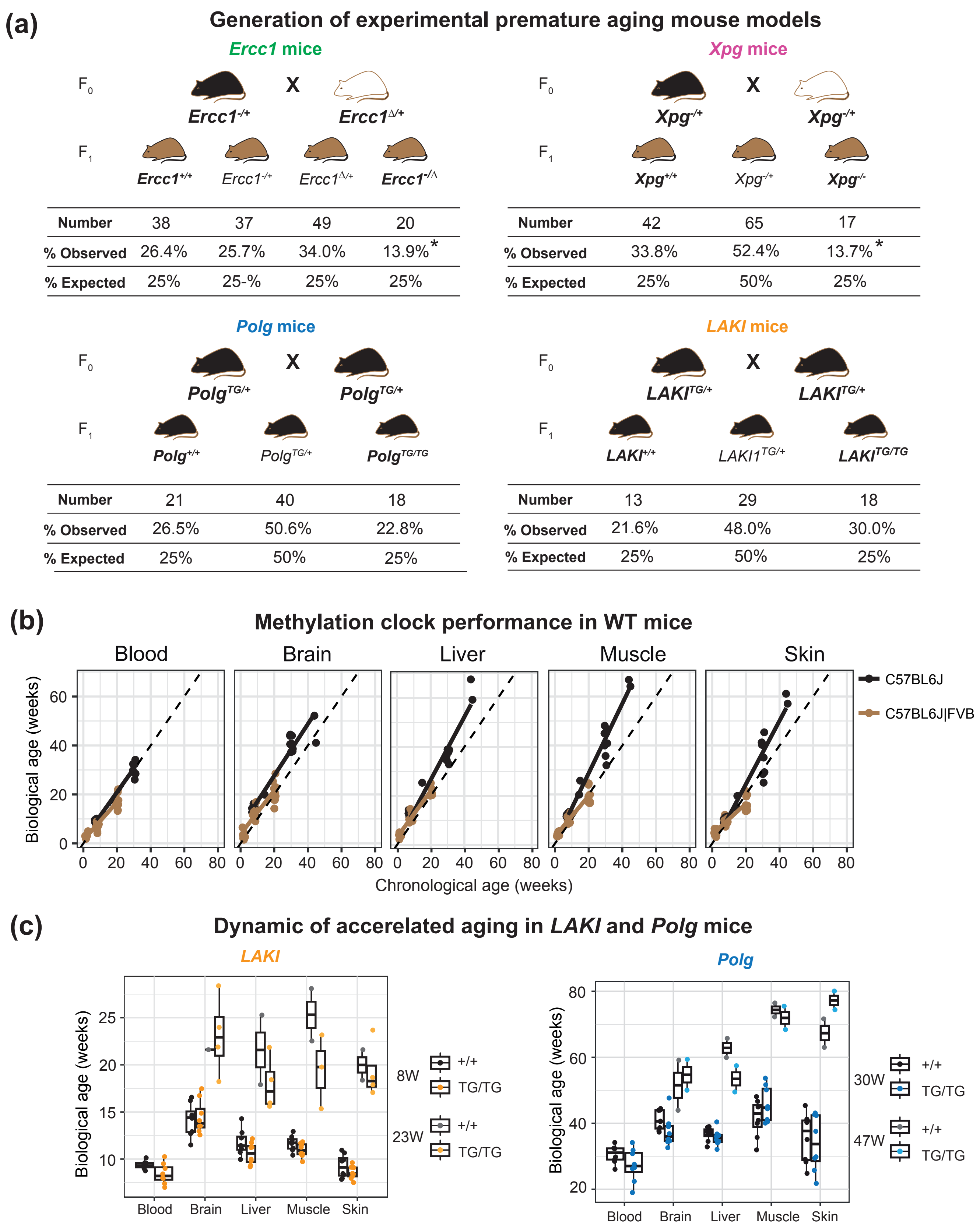

Figure S1. DNA methylation in premature aging mouse models additional data. (a) Breeding protocol to generate the four premature mouse strains and littermate control mice. Statistical significance was assessed by Pearson's chi-squared test. Background C57BL6J (Black), FVB (white), C57BL6J | FVB hybrid (brown). (b) Correlation between biological and chronological age (in weeks) in WT control mice in C57BL6J and C57BL6J|FVB backgrounds in analyzed tissues from 2- to 47-week-old. (c) Methylation biological age of *Polg*$^{TG/TG}$ (at 30 and 47 weeks old) and *LAKI*$^{TG/TG}$ (at 8 and 23 weeks) in multiple organs/tissues and WT littermate controls by Horvath clock. Data are represented as box plots (center line shows median, box shows 25th and 75th percentiles and whiskers show minimum and maximum values) and statistical significance was assessed by two-sided unpaired t-test.

Tissue	C57BL6J [RMSE (r)]	C57BL6J | FVB [RMSE (r)]
**Blood**	2.08 (0.99)	2.55 (0.95)
**Brain**	8.71 (0.96)	4.13 (0.89)
**Liver**	8.49 (0.98)	3.04 (0.97)
**Muscle**	11.2 (0.98)	2.51 (0.95)
**Skin**	7.59 (0.96)	3.21 (0.91)

Table S1. Horvath methylation clock performance in multiple tissues in control WT mice

Model	Timepoint	Tissue	WT [Avg (Sd; N)]	KO [Avg (Sd; N)]	P (t-test)	Sig
Ercc1	2w	Blood	3.17 (0.93; 8)	4.35 (0.86; 7)	0.025	*
		Brain	3.37 (1.53; 8)	3.12 (0.84; 8)	0.69	
		Liver	4.05 (0.9; 8)	4.89 (0.92; 7)	0.098	
		Muscle	3.86 (0.88; 8)	4.75 (1.33; 8)	0.138	
		Skin	4.33 (1.25; 8)	4.91 (1.25; 8)	0.368	
	8w	Blood	6.85 (1.62; 8)	12.46 (1.08; 8)	0	***
		Brain	12.8 (1.29; 8)	15.01 (1.84; 8)	0.016	*
		Liver	11.66 (1.33; 8)	14.78 (1.49; 8)	0.001	**
		Muscle	9.98 (1.23; 8)	11.87 (1.1; 8)	0.006	**
		Skin	9.68 (1.36; 8)	11 (1.06; 8)	0.049	*
	20w	Blood	16.95 (2.95; 10)	27.58 (3.83; 6)	0	***
		Brain	20.7 (4.23; 10)	25.39 (2.54; 6)	0.015	*
		Liver	21.56 (1.74; 9)	25.07 (3.62; 6)	0.065	
		Muscle	19.2 (2.95; 10)	25.82 (3.69; 6)	0.005	**
		Skin	15.97 (3.04; 10)	18.91 (2.37; 6)	0.051	
Xpg	8w	Blood	7.96 (0.73; 8)	9.09 (1.08; 8)	0.03	*
		Brain	12.3 (2.4; 8)	13.67 (1.58; 8)	0.201	
		Liver	11.11 (1.83; 8)	11.45 (1.54; 8)	0.702	
		Muscle	10.01 (1.8; 8)	10.07 (2.04; 8)	0.947	
		Skin	8.79 (1.14; 8)	8.18 (0.7; 8)	0.22	
LAKI	8w	Blood	9.35 (0.42; 8)	8.44 (1.09; 8)	0.054	
		Brain	14.17 (1.74; 8)	14.46 (1.78; 8)	0.745	
		Liver	11.74 (1.34; 8)	10.59 (1.15; 8)	0.086	
		Muscle	11.66 (0.81; 8)	10.94 (0.74; 8)	0.085	
		Skin	9.24 (1.24; 8)	8.55 (0.69; 8)	0.199	
	23w	Brain	19.87 (NA; 1)	21.27 (3.54; 4)		
		Liver	21.13 (6; 2)	16.97 (2.31; 4)	0.501	
		Muscle	22.78 (4.04; 2)	17.92 (3.91; 3)	0.303	
		Skin	17 (3.36; 2)	15.92 (2.2; 4)	0.733	
Polg	30w	Blood	30.6 (2.68; 8)	27.3 (4.99; 8)	0.129	
		Brain	40.89 (2.93; 8)	37.5 (4.76; 8)	0.113	
		Liver	36.81 (2.15; 8)	35.63 (2.44; 8)	0.322	
		Muscle	41.67 (5.64; 8)	45.81 (5.36; 8)	0.154	
		Skin	35.6 (7.44; 8)	34.05 (8.42; 8)	0.702	
	47w	Brain	46.62 (7.83; 2)	45.96 (6.12; 2)	0.934	
		Liver	63.41 (5.88; 2)	51.25 (7.35; 2)	0.215	
		Muscle	65.48 (1.9; 2)	61.37 (6.16; 2)	0.512	
		Skin	59.1 (2.87; 2)	63.98 (1.8; 2)	0.202	

Table S2: Horvath methylation clock performance in multiple tissues in primature aging mouse models.
