# Supplementary material for: DNA repair‐deficient premature aging models display accelerated epigenetic age
Source: Aging Cell. 2023 Dec 22;23(2):e14058. doi: 10.1111/acel.14058 (PMC10861193; doi:10.1111/acel.14058)
Supplement: Supplementary file 1 — Appendix S1 [file ACEL-23-e14058-s001.zip › acel14058-sup-0002-AppendixS2.pdf]

Supplementary Material

Extended Methods

Animal housing

All the animal experiments were performed in accordance with Swiss legislation after the approval from the local authorities (Cantonal veterinary office, Canton de Vaud, Switzerland). Mice were housed in groups of five per cage with a 12hr light/dark cycle between 06:00 and 18:00 in a temperature-controlled environment at 25°C and humidity between 40 % and 70 %, with free access to water and food. Wild type (WT) and premature aging mouse models used in this study were generated by breeding (Figure S1A) and housed together until they reached the desired age in the Animal Facilities of Epalinges and Department of Biomedical Science of the University of Lausanne.

Mouse strains

Ercc1$^{-/\Delta}$ [1] and *Xpg*$^{-/-}$ mice [2] and littermate controls (*Ercc1*$^{+/+}$ and *Xpg*$^{+/+}$) were used in C57BL6J|FVB hybrid background. *Polg*$^{D257A/D257A}$, herein referred to as Polg$^{TG/TG}$ [3,4] and LAKI$^{TG/TG}$ [5] and sibling controls (Polg$^{+/+}$ and LAKI$^{+/+}$) were generated in C57BL6J background.

Mouse monitoring and euthanasia

All mice were monitored at least three times per week to evaluate their activity, posture, alertness, body weight, presence of tumors or wound, and surface temperature. Males and females were euthanized at the specific timepoints by CO_2 inhalation (6 min, flow rate 20% volume/min). Subsequently, before perfusing the mice with saline, blood was collected from the heart. Finally, multiple organs and tissues were collected in liquid nitrogen and used for DNA extraction to perform MethylArray.

Cell culture and maintenance

Human fibroblasts were obtained from the Coriell cell repositories and cultured in DMEM (Gibco, 11960085) with 10% FBS (Hyclone, SH30088.03) containing non-essential amino acids, GlutaMax and Sodium Pyruvate (Gibco, 11140035, 35050061, 11360039) at 37°C in hypoxic conditions (3% O_2). Subsequently, fibroblasts were passaged and cultured according to standard protocols.

DNA extractions

Total DNA was extracted from tissues and cells using Monarch Genomic DNA Purification Kit (New England Biolab, T3010L) and protocols were carefully followed. Tissues were cut into small pieces to ensure rapid lysis. Total DNA concentrations were determined using the Qubit DNA BR Assay Kit (Thermofisher, Q10211).

DNA methylation clock

The mouse clock was developed in Mozhui et al. [6]. The "pan-tissue" and tissue-specific mouse clocks were used for the analysis of different tissues. For the analysis of fibroblasts, the "Skin&Blood" clock was used, as this has previously been shown to be more accurate than the "pan-tissue" clock to assess age of human fibroblasts [10]. The software code of the mouse clocks can be found in the supplements of [6]. The mouse methylation data were generated on the small and the extended version of HorvathMammalMethylChip [7]. We used the SeSaMe normalization method [8]. Human methylation data were generated on the Illumina EPIC array platforms that profiles 866k cytosines. We used the noob normalization method implemented in the R function preprocessNoob. The human DNAm age was estimated using the Skin&blood clock algorithm [9].

Statistical analysis

Unsupervised hierarchical clustering based on interarray correlation coefficients was used to identify putative technical outliers. One liver sample with negative methylation age was removed. All plots were generated using the R software package ggplot2. Statistical differences between groups were assessed using a two-tailed unpaired Student's t-test. Clock performance was assessed by correlation (Pearson coefficient) and Random Mean Square Error (RMSE), using the R software. To determine if there was a significant difference in the slope of aging between WT and transgenic mice, we looked at the significance of the interaction term in the linear regression: DNAm age ~ WT/TG + Age + WT/TG*Age.

1 de Waard, M. C. *et al.* Age-related motor neuron degeneration in DNA repair-deficient Ercc1 mice. *Acta Neuropathol* **120**, 461-475, doi:10.1007/s00401-010-0715-9 (2010).

2 Barnhoorn, S. *et al.* Cell-autonomous progeroid changes in conditional mouse models for repair endonuclease XPG deficiency. *PLoS Genet* **10**, e1004686, doi:10.1371/journal.pgen.1004686 (2014).

3 Kujoth, G. C. *et al.* Mitochondrial DNA mutations, oxidative stress, and apoptosis in mammalian aging. *Science* **309**, 481-484, doi:10.1126/science.1112125 (2005).

4 Trifunovic, A. *et al.* Premature ageing in mice expressing defective mitochondrial DNA polymerase. *Nature* **429**, 417-423, doi:10.1038/nature02517 (2004).

5 Osorio, F. G. *et al.* Splicing-directed therapy in a new mouse model of human accelerated aging. *Sci Transl Med* **3**, 106ra107, doi:10.1126/scitranslmed.3002847 (2011).

6 Mozhui, K. *et al.* Genetic loci and metabolic states associated with murine epigenetic aging. *Elife* **11**, doi:10.7554/eLife.75244 (2022).

7 Arneson, A. *et al.* A mammalian methylation array for profiling methylation levels at conserved sequences. *Nat Commun* **13**, 783, doi:10.1038/s41467-022-28355-z (2022).

8 Zhou, W., Triche, T. J., Jr., Laird, P. W. & Shen, H. SeSAMe: reducing artifactual detection of DNA methylation by Infinium BeadChips in genomic deletions. *Nucleic Acids Res* **46**, e123, doi:10.1093/nar/gky691 (2018).

9 Horvath, S. *et al.* Epigenetic clock for skin and blood cells applied to Hutchinson Gilford Progeria Syndrome and ex vivo studies. *Aging (Albany NY)* **10**, 1758-1775, doi:10.18632/aging.101508 (2018).

10 Horvath S, Oshima J, Martin GM, Lu AT, Quach A, Cohen H, Felton S, Matsuyama M, Lowe D, Kabacik S, Wilson JG, Reiner AP, Maierhofer A, Flunkert J, Aviv A, Hou L, Baccarelli AA, Li Y, Stewart JD, Whitsel EA, Ferrucci L, Matsuyama S, Raj K. Epigenetic clock for skin and blood cells applied to Hutchinson Gilford Progeria Syndrome and ex vivo studies. Aging (Albany NY). 2018 Jul 26;10(7):1758-1775. doi: 10.18632/aging.101508. PMID: 30048243; PMCID: PMC6075434.
