# Supplementary material for: DNA repair‐deficient premature aging models display accelerated epigenetic age
Source: Aging Cell. 2023 Dec 22;23(2):e14058. doi: 10.1111/acel.14058 (PMC10861193; doi:10.1111/acel.14058)
Supplement: Supplementary file 1 — Appendix S1 [file ACEL-23-e14058-s001.zip › captions.docx]

Table S3: Metadata for human samples.

Table S4: Methylation biological age (years) in multiple tissues of premature aging mouse models.
